# Preparing Europe for future health threats and crises – key elements of the European Centre for Disease Prevention and Control’s reinforced mandate

**DOI:** 10.2807/1560-7917.ES.2023.28.3.2300033

**Published:** 2023-01-19

**Authors:** Maarit Kokki, Andrea Ammon

**Affiliations:** 1 European Centre for Disease Prevention and Control (ECDC), Stockholm, Sweden

**Keywords:** public health, European Health Union, health threats, health emergency, crisis, preparedness

The COVID-19 pandemic has taught us many lessons and several of these have now been captured in European Union (EU) legislation. In order to be better prepared for future health threats and crises, the mandates of the European Centre for Disease Prevention and Control (ECDC) [1] and the European Medicines Agency (EMA) [2] have been reinforced, the European Commission’s Directorate General European Health Emergency Preparedness and Response Authority (HERA) [3] was created and the Regulation on serious cross border threats to health came into force [4]. The lessons from the past 3 years also have implications for public health preparedness beyond the current pandemic, and many of them are transferable to other cross-border health threats, such as the risk of the emergence of new pathogens, resistance to antimicrobials and to vaccine preventable diseases.

ECDC’s reinforced mandate [1], was adopted by the European Parliament on 4 October 2022 and by the Council on 24 October 2022. It further strengthens the capacity of ECDC to provide the robust and independent scientific expertise necessary to support actions related to the prevention, preparedness, and response planning to prevent and control serious cross border threats to health in accordance with Regulation (EU) 2022/2371 [4]. Since 26 December 2022, ECDC is mandated to provide non-binding science-based recommendations setting out options for both the management and control of communicable diseases in humans and related special health issues. ECDC will work closely with the EU countries to determine how to effectively monitor the capacity of their health systems to detect, prevent, respond, and recover from outbreaks. Further, the Centre will provide science-based recommendations to strengthen the health systems in the EU countries, and to support them in the implementation of projects to fill in any identified gaps.

Secure and interoperable digital platforms and applications in support of epidemiological surveillance are crucial at EU level. Following its reinforced mandate, ECDC has the task to develop such systems and it will seek solutions that allow a better use of the data by applying new digital technologies, such as artificial intelligence and computer modelling in data compilation and analysis.

Improving epidemiological surveillance through digitalisation of integrated surveillance systems in the EU countries, will constitute the basis for an EU-level digitalised surveillance system of communicable diseases, with interoperability across borders. Interconnected systems would ease monitoring of the impact of communicable disease crises (e.g. pandemics) on the healthcare system/hospitals in the future. The enhanced capacity to obtain a comprehensive overview of the epidemiological situation at EU level, to monitor the impact of communicable disease events on the healthcare system, and the possibility to anticipate future trends should be the EU-added value of surveillance of which the outcomes will be made available to decision-makers at EU and national levels.

Further, future-oriented information to decision-makers at EU and national levels will be provided through an ECDC programme on epidemiological modelling, anticipation, and scenario development for response. Based on the future scenarios, this programme will support prioritisation and preparedness at ECDC-, EU-, and country level. ECDC will also support and complement similar efforts of other EU entities, including HERA’s intelligence gathering and threat assessment activities, towards a more resilient EU. ECDC will leverage its biostatistics, disease modelling, and health economics capacity to provide in-depth analyses, risk assessments, and recommendations for policy and actions to prevent and control communicable diseases and other special health issues.

In addition to the current network for epidemiological surveillance, ECDC will operate and coordinate two new networks: the network of EU reference laboratories for public health, and a network of national services supporting the use of substances of human origin.

To provide hands-on technical support to the EU countries and to third countries in outbreak situations, or to support countries in their preparedness and response planning, ECDC will also establish a permanent EU Health Task Force (EUHTF) (Box). The EUHTF will consist of an ECDC team coordinating the set up and future routine operations. An Enhanced Emergency Capacity will be composed of EUHTF public health experts from EU/EEA (European Economic Area) countries on voluntary basis, ECDC experts, and fellows during their two-year placement in the ECDC Fellowship Programme. They will support outbreak investigations or preparedness and response activities using their knowledge and practice related to preparedness and response during deployments and, to the benefit of their own countries, when not deployed. To enable smooth deployments and logistics support in particular in third countries, ECDC will establish arrangements with the EC Directorate General for European Civil Protection and Humanitarian Aid Operations and with the Global Outbreak Alert and Response Network.

Box **Scope of the European Union Health Task Force**

**Timely emergency response during outbreaks and crises**

Communicable diseases or diseases of unknown origin;
Remote support and rapid in-country field deployment;
Outbreak investigation/response, operational research, guidance, resources, and tools.

**Strengthening countries emergency preparedness**

Development, testing, and updating of preparedness protocols and plans;
Assessment of preparedness gaps;
Simulation exercises and in- and after-action reviews;
Tailored capacity building activities and training.

As part of the EU commitment to reinforce partners’ preparedness and response capacity to communicable diseases, laid out in the recent EU Global Health Strategy [5], as well as through the amended mandate, ECDC will focus on wider global health considerations. The Centre will cooperate with public health actors in third countries, and international organisations competent in the field of public health.

ECDC is one of the building blocks of the new European Health Union, together with EMA and HERA. Coordination of work between the two EU agencies and the EC, including HERA, and the World Health Organization Regional Office for Europe is of utmost importance to avoid duplication of actions, enhance efficiency and effectiveness, and create synergy.

During the COVID-19 pandemic, the coordination mechanisms between the EC and ECDC have been further strengthened. The mandates of HERA and ECDC are well defined; HERA provides solutions to pandemic preparedness and response by focusing on the whole value chain of medical countermeasures and ECDC is tasked to identify, assess, and communicate current and emerging threats from communicable diseases and related special health issues. With the reinforced mandate, ECDC is also tasked to monitor the prevention, preparedness and response plans of countries and support them in addressing identified gaps. Some of ECDC and HERA’s areas of work are closely related and therefore alignment of actions and collaboration are necessary. ECDC and HERA will sign a Memorandum of Understanding outlining areas of enhanced collaboration and complementary actions.

ECDC and EMA have been working together since 2010 through a bilateral collaboration agreement. Over the years of close collaboration, the respective responsibilities and complementary areas of work have become clear. Joint work on the development of a post-authorisation vaccine monitoring platform goes back almost 10 years, and now with reinforced mandates this jointly operated platform became reality. Independent studies on effectiveness, safety, and the use of vaccines carried out through this joint vaccine monitoring platform will provide information at EU and national levels on how vaccines perform in real life. This will help national authorities to make decisions on immunisation programmes, which is essential to build and maintain trust of the general population in vaccines.

Looking ahead, there is a growing awareness that societies should be more alert to the impact of communicable disease outbreaks, as well as to the impact public health countermeasures might have on other sectors of society. ECDC’s reinforced mandate bolsters the agencies’ possibilities to look at the interconnectivity of communicable diseases, non-communicable diseases, and health determinants. The next external evaluation of ECDC is foreseen in 2025. It will assess the possibility to expand ECDC’s mandate to non-communicable health threats as well as the degree of the implementation of the reinforced mandate.

To implement the reinforced mandate, ECDC will enhance its ways of working both internally and with external partners. It will develop even closer relations with the EU countries to understand their context better and to set a joint level of ambition for the implementation of the legislation. ECDC will foster cooperation, take the lead when necessary, and support countries in every feasible way. However, for the large transformations such as building national digitalised integrated surveillance systems as prerequisites for an EU level system, coordinated efforts at different levels are necessary.

Through collaborative actions between ECDC, EU countries, other EU Agencies, and the EC, the new European Health Union which is built on solidarity and equity, could decrease health inequalities.

One of the main lessons from the COVID-19 pandemic for future health emergency preparedness planning is that we need to adopt a multi-sectorial and multi-disciplinary preparedness approach, with ‘One Health’ in focus and where we also acknowledge globalisation and climate change as drivers of risk. Experiences made during the past years need to be used to improve our surveillance systems and build capacity and resilience in the public health systems to be better prepared for future health crises in the 21st century. It is crucial that lessons learned lead to continued improvement and adaptation. To ensure this, continuous political will and sustained investment in public health at national and at EU level are needed.

## Note

Maarit Kokki is Head of the Executive Office at the European Centre for Disease Prevention and Control (ECDC) and Andrea Ammon is the Director of ECDC.
